# Experimental autoimmune encephalomyelitis from a tissue energy perspective

**DOI:** 10.12688/f1000research.11839.1

**Published:** 2017-11-08

**Authors:** Roshni A Desai, Kenneth J Smith

**Affiliations:** 1 Department of Neuroinflammation, UCL Institute of Neurology, London, UK

**Keywords:** multiple sclerosis, Experimental autoimmune encephalomyelitis, tissue energy metabolism

## Abstract

Increasing evidence suggests a key role for tissue energy failure in the pathophysiology of multiple sclerosis (MS). Studies in experimental autoimmune encephalomyelitis (EAE), a commonly used model of MS, have been instrumental in illuminating the mechanisms that may be involved in compromising energy production. In this article, we review recent advances in EAE research focussing on factors that conspire to impair tissue energy metabolism, such as tissue hypoxia, mitochondrial dysfunction, production of reactive oxygen/nitrogen species, and sodium dysregulation, which are directly affected by energy insufficiency, and promote cellular damage. A greater understanding of how inflammation affects tissue energy balance may lead to novel and effective therapeutic strategies that ultimately will benefit not only people affected by MS but also people affected by the wide range of other neurological disorders in which neuroinflammation plays an important role.

## Introduction

Multiple sclerosis (MS) is an immune-mediated disease of the central nervous system (CNS), characterised by multifocal, perivenous inflammation and focal destruction of myelin, typically resulting in a relapsing-remitting pattern of neurological deficit and leading to a progressive, neurodegenerative pathology. MS is a heterogeneous disease, with a multifactorial aetiology ^1– 4^ and a highly variable clinical course. It is therefore perhaps not surprising that the treatment of MS remains complex. The sequence of events in lesion genesis remains uncertain, particularly the genesis of the first lesion, and different patterns of lesion formation have been distinguished ^5^, making modelling of the disease in animals difficult. Although no single model reflects the entire clinical and pathological spectrum of MS ^6^, over the years autoimmune ^7^, toxic ^8, 9^ and viral ^10^ models have all been employed to understand different aspects of the disease. However, experimental autoimmune encephalomyelitis (EAE) has become the most widely used laboratory model of MS, and it has various factors in common with MS, including genetic susceptibility, age, and gender, influence the clinical course, and pathology of EAE, together with the type and preparation of antigen employed and the dose and route of administration ^7, 11, 12^. Though criticised as a model of MS ^13, 14^, EAE remains a good model of CNS inflammation, and several treatments, including glatiramer acetate ^15, 16^, fingolimod ^17, 18^ and natalizumab ^19^, have been successfully translated from EAE to MS. Thus, if used wisely, EAE can be a valuable tool for better understanding not only the pathophysiology of acute MS-like lesions but also the mechanisms involved in dysfunction, damage and progression in MS in order to identify novel therapeutic targets.

In addition to implicating inflammation and demyelination, pathological studies have implicated neuronal and axonal damage and loss, which begin early in the course of the disease, as major causes of irreversible disability in patients with MS ^20, 21^. Although the exact mechanisms associated with irreversible neuronal and axonal loss in MS and EAE are poorly understood and probably multifactorial, mitochondrial dysfunction ^22– 28^ and subsequent energy insufficiency are increasingly recognised as important contributing factors ^29– 31^. In this review, we summarise the most recent advances in EAE research from an energy viewpoint, and focus on tissue hypoxia, mitochondrial dysfunction, reactive oxygen/nitrogen species (ROS/RNS) and sodium dysregulation.

## Energy production in the central nervous system

The brain is a highly metabolic organ: whilst comprising only 2% of the body’s mass, it requires 20% of the body’s resting energy consumption ^32^. Neurons have the highest energy demand ^33^, and it has been hypothesised that these energetic demands are met in part by energy production in neighbouring glia ^34– 36^. Glucose has often been considered the major metabolite used during oxidative phosphorylation for yielding energy in neurons and glia, but in fact different cell types can preferentially use different metabolic substrates and pathways to produce ATP under physiological conditions and, besides glucose, neurons and glial cells, can use lactate, pyruvate, glutamate and glutamine as metabolic substrates ^37^. Oxidative phosphorylation yields approximately 30 molecules of ATP per molecule of glucose, compared with a meagre two molecules of ATP per molecule of glucose via glycolysis ^38^. Therefore, although ATP production by glycolysis is rapid, it is understandable why oxygen is essential to ensure an efficient energy metabolism, particularly in metabolically demanding cells such as neurons. Indeed, neurons demand a considerable supply of energy, and mitochondria, the key providers of this energy, must be distributed in appropriate numbers to meet demand.

Theoretical energy budgets have established that the majority of the energy consumed by the brain is used for restoration and maintenance of the resting membrane potential by the sodium-potassium (Na-K) ATPase ^33, 39, 40^, especially following electrical activity. However, a significant proportion of the brain’s energy expenditure is associated with non-signalling or ‘housekeeping’ processes, including lipid turnover, proton leak across the mitochondrial membrane, cytoskeletal rearrangements, vesicle recycling, and protein synthesis ^40, 41^. Therefore, it is easy to imagine how an impairment of energy metabolism can have significant consequences on CNS function. A number of factors, including nitric oxide (NO), other ROS/RNS and factors from immune cells, and tissue hypoxia, have been suggested to conspire to impair ATP production by compromising mitochondrial function in MS ^42– 45^. Deficiencies in mitochondrial transport and the mitochondrial respiratory chain enzymes, notably complex IV, will also impair ATP production, and the nature and importance of these deficiencies may well change during the different phases of the disease.

## Consequences of energy failure

Energy failure due to mitochondrial dysfunction or damage is increasingly recognised to play a pivotal role in MS pathogenesis. Mitochondrial defects, which will certainly impair ATP production, have been demonstrated within acute ^43^ and chronic ^28, 46, 47^ lesions and also in the normal appearing white matter ^22^ of patients with MS. Indeed, metabolites produced as a probable consequence of ATP depletion are observed in the cerebrospinal fluid of patients with MS ^48^. Impaired ATP production can reduce sodium extrusion from the axoplasm into the extracellular space following electrical activity, and this deficit will be exaggerated in conducting demyelinated axons because of their increased expression and redistribution of sodium channels along the demyelinated axolemma ^49^ associated with the restoration of electrophysiological function ^50– 52^. The excessive accumulation of intracellular sodium ions not only increases the energy demand to operate the Na-K ATPase, which is already one of the most significant ATP consumers in the CNS ^53^, but it also promotes the reverse operation of the sodium-calcium exchanger (NCX) ^54, 55^, which imports calcium into the axoplasm. The energy-starved axon is unable to restore calcium homeostasis, resulting in calcium cytotoxicity and the initiation of cell death pathways. Such energy failure within axons further increases their susceptibility to excitotoxic injury ^56^. Small-diameter fibres, which preferentially degenerate in MS, may be more vulnerable to energy failure than their larger neighbours because of their lower mitochondrial number in relation to their surface area ^57^, although other mitochondrially mediated mechanisms may also play a role ^58^.

Thus, there is good evidence that energy failure may play an important role in axonal degeneration in MS, and it follows that neuroprotective strategies aimed at protecting energy balance may be effective in MS. The animal model of MS, EAE, has been employed not only to study the role of energy deficits but also to explore strategies to achieve neuroprotection.

## Tissue hypoxia in experimental autoimmune encephalomyelitis

Increasing evidence from neuropathological and magnetic resonance imaging (MRI) studies shows that MS lesions can experience low oxygen concentrations ^59– 61^. Hypoxia can decrease mitochondrial oxygen consumption in cells (the respiratory rate) ^62^ by initiating a decrease in ATP-using processes (metabolic demand) and also can have significant effects on mitochondrial movement, velocity and morphology; however, it is unclear from the human data alone whether the tissue hypoxia contributes to the pathogenesis of the disease. The presence of severe tissue hypoxia sufficient to compromise function was demonstrated by Davies *et al*. ^63^, who showed that the neurological deficits in a rat model of EAE quantitatively, spatially and temporally correlated with spinal white and grey matter hypoxia. Two independent methods were used to demonstrate hypoxia: an intravenous immunohistochemical ^64^ probe and a fine, oxygen-sensitive optical probe physically inserted into the spinal cord. The authors demonstrated the importance of hypoxia in the expression and progression of neurological deficits by showing that treatment with both acute (1 hour) and prolonged (7 continuous days) normobaric oxygen (~95%) reversed the hypoxia and partially restored function and attenuated disease severity, respectively. More recently, hypoxia was found to be a key factor in lesion formation in an animal model of the pattern III demyelinating lesion found in MS ^65^. This experimental lesion is induced by the intraspinal injection of the pro-inflammatory agent lipopolysaccharide into the dorsal white matter of adult rats ^66^, and the authors found that the demyelination was reduced, or even prevented, by breathing normobaric oxygen during the two-day period when the spinal cord otherwise would have been hypoxic ^65^. These findings may recommend the consideration of oxygen therapy for acute attacks in MS, but if extrapolation is considered to include progressive disease it is important to bear in mind that mitochondrial respiratory chain enzymes are deficient within neurons and that mitochondrial transport is also likely to be impaired, and these considerations may limit the therapeutic potential of oxygen therapy. Thus, the cause or causes of damage in progressive disease are not necessarily the same (and are not likely to be precisely the same) as those in the acute lesion.

A role for hypoxia in MS was further supported by Johnson *et al*. ^67^, who measured oxygenation in the cerebellum and cortex of awake, unrestrained mice with EAE and found that the grey matter was severely hypoxic. The same group also used susceptibility-weighted imaging (SWI) to assess deoxyhaemoglobin-based hypointensities in EAE mice *in vivo* ^68^. SWI is a protocol with MRI that is particularly sensitive to deoxyhaemoglobin and can visualise the venous vasculature ^69, 70^, but it also detects parenchymal iron deposits and demyelination ^71, 72^, which complicates the interpretation when imaging patients with MS and animals with EAE. Given that the visibility of the venous vasculature is highly dependent on the partial pressure of oxygen, Nathoo *et al*. ^68^ modulated the inspired oxygen concentration during imaging, hypothesising that vascular hypointensities visible with normoxic conditions would disappear upon an increase in inspired oxygen. The authors concluded the presence of venous hypoxia due to the increased oxygen demand, arising from inflammation, outstripping supply ^68^.

Esen *et al*. ^73^ adopted a different approach, namely exposing mice to normobaric *hypo*xia for three weeks from the day of immunisation for EAE, to induce angioplasty and tissue survival. This strategy not only significantly delayed the onset of disease but also decreased inflammatory activity in the spinal cords of the mice. The authors attributed the beneficial effects to the induction of an anti-inflammatory milieu, but hypoxic pre-conditioning of the tissue to survive the hypoxic insult associated with EAE ^63, 67^ may also have played a role.

## Reactive oxygen/nitrogen species in experimental autoimmune encephalomyelitis

ROS/RNS are routinely produced under physiological conditions; however, they normally pose very little threat because of a specialised set of endogenous defence and repair mechanisms. The CNS anti-oxidant defence system is composed of non-enzymatic (for example, glutathione [GSH] and uric acid) and enzymatic (for example, superoxide dismutases, GSH peroxidase, catalase, haeme-oxygenases, quinone oxidoreductases and peroxiredoxins) anti-oxidants ^74, 75^. During pathological conditions, such as inflammation, the overproduction of ROS/RNS overwhelms this anti-oxidant system, resulting in oxidative/nitrative stress. ROS/RNS and the ensuing oxidative/nitrative stress have frequently been suggested to play an important early role in MS ^44, 45^ and EAE ^76^, mainly through their toxic actions on mitochondria ^76, 77^, and therefore can indirectly contribute to a tissue energy deficit. ROS/RNS include superoxide, peroxynitrite and the hydroxyl radical. Superoxide, produced by a one-electron reduction of oxygen, is the precursor of most other forms of ROS. Dismutation of superoxide produces hydrogen peroxide, which in turn either can be fully reduced to water or, in the presence of ions of a suitable transition metal (for example, iron), can lead to the formation of the extremely toxic hydroxyl radical ^78^. Superoxide can also react with NO, in a reaction that is limited by the rate of diffusion of both radicals, to produce peroxynitrite. Mitochondria themselves produce low levels of superoxide under normal conditions; however, superoxide production can increase significantly under pathological conditions, particularly when mitochondria are damaged, or if the cytoplasmic oxygen concentration is abnormally high or low ^79^. Nevertheless, the most abundant source of superoxide is the respiratory burst, which is mediated by the nicotinamide adenine dinucleotide phosphate (NADPH) oxidases ^80^. Mossakowski *et al*. ^81^, using intravitral NADPH fluorescence lifetime imaging to detect functional NADPH oxidase (NOX) enzymes *in vivo*, recently reported a spatio-temporal correlation between the activated NOX enzymes and neuronal damage in mice with EAE. The authors identified activated macrophages/microglia as major cellular sources of activated NOX enzymes but showed for the first time that astrocytes are also major contributors of oxidative stress in the CNS during chronic EAE. As NOX activation was not restricted to a specific cell type, Mossakowski *et al*. suggested that a locally acting soluble mediator, such as glutamate, may contribute to the activation seen, and they supported this by applying glutamate locally and measuring the increase in NOX enzyme activation in the brain stem of healthy mice. The authors observed excessive ROS production and a concomitant increase in neuronal calcium. Besides CNS NOX activity, NOX enzyme was overactivated in peripheral CD11b ^+^ monocytes from mice with EAE and patients with relapsing-remitting MS, an effect that can be antagonized by systemic administration of epigallocatechin-3-gallate, the major polyphenolic compound of the green tea plant ^81^. Recently, Radbruch *et al*. ^82^ used the same technique to investigate whether NOX activity was evident during the remission phase of EAE. They found that during this phase of the disease, when neurological function in the mice is restored, astrocytes and microglia shift towards an activated phenotype, showing morphological changes and elevated levels of activated NOX enzymes, which correlated with subclinical neuronal dysfunction, characterised by elevated neuronal calcium.

Besides changes in NOX activity, alterations in other mediators of oxidative stress have been revealed in EAE. Two independent groups have reported reduced GSH levels in rodents with clinical EAE ^75, 83^. Morales Pantoja *et al*. ^83^ found that this reduced GSH is a consequence of decreased levels of the enzymes and transporters that are required for *de novo* GSH synthesis because of diminished levels of nuclear factor (erythroid-derived 2)-like 2 (Nrf2) in EAE. Nrf2 is a transcription factor that regulates endogenous anti-oxidant systems. Under conditions of oxidative stress, Nrf2 translocates to the nucleus to promote the expression of Nrf2-regulated genes, including those that are involved in GSH synthesis. Nrf2 was low in both the cytoplasmic and nuclear fractions; however, the decrease in the latter was more severe, suggesting that the nuclear transport of Nrf2 is also affected in EAE ^83^. Interestingly, dimethyl fumarate, a current therapy for patients with MS, increases Nrf2 ^84^, thus increasing GSH levels.

## Mitochondrial dysfunction in experimental autoimmune encephalomyelitis

Several studies have reported significant mitochondrial abnormalities in both MS ^22, 43, 46, 47^ and EAE ^76, 77, 85^. Indeed, mitochondrial alterations such as mitochondrial vacuolisation, swelling and dissolution of cristae have been described to occur as early as three days post-immunisation of EAE ^76^. Early morphological alterations in mitochondria are associated with focal axonal damage in axons with intact myelin ^77^. Furthermore, whereas large-calibre axons recovered from such damage, thin-calibre axons had higher rates of progression ^77^.

Sadeghian *et al*. ^85^ recently showed that mitochondria in spinal cord axons of EAE mice are depolarised, often totally so, and fragmented, and have impaired trafficking. The mitochondrial deficits correlated with the neurological deficit, reversing during remission and re-occurring at relapse. The authors showed that this mitochondrial dysfunction was most severe within inflammatory foci but independent of demyelination or degeneration ^85^. The data implicate mitochondrial dysfunction and energy failure as major causes of neurological deficits in the absence of overt structural damage.

Dysfunctional mitochondria usually generate more ROS, promoting increased oxidative stress. Accordingly, subtle decreases in mitochondrial membrane potential, coupled with increased oxidative stress, have been described prior to the onset of neurological deficit and immune cell infiltration in EAE ^86^. In addition to decreases in mitochondrial membrane potential, certain conditions such as oxidative stress, low adenine nucleotide concentrations, or increased calcium induce the formation of the mitochondrial permeability transition pore (mPTP) ^87, 88^. Opening of this pore, as suggested by its name, increases the permeability of the mitochondrial membranes to low-molecular-weight solutes and some proteins, and it is one of the main causes of cell death. The peptidylprolyl *cis-trans*-isomerase cyclophilin D (CypD) is considered critical for opening the mPTP ^89, 90^, and it was recently shown that a mitochondrially targeted CypD inhibitor significantly improved neurological deficit and protected axons, with minimal immunosuppression, in murine EAE ^91^.

## Sodium dysregulation in experimental autoimmune encephalomyelitis

One of the main consequences of energy failure is sodium dysregulation (that is, an excessive accumulation of sodium in axons) due to an inadequacy of sodium extrusion. A number of studies have implicated a role for voltage-gated sodium channels (VGSCs), and a rise in intra-axonal sodium ions, in promoting the degeneration of myelinated axons. A rise in internal sodium is not usually problematic if there is adequate ATP to restore sodium homeostasis via the Na-K ATPase (sodium pump), but energy insufficiency can allow sodium to rise sufficiently to cause reverse operation of the NCX and the importation of lethal quantities of calcium ions. This is particularly the case in demyelinated axons, which are more vulnerable to sodium dysregulation because of their adaptive re-expression of sodium channels along the denuded axolemma. Important early studies were performed in optic nerve axons with energy failure due to imposed anoxia (for example, 92), but the recognition that NO both was a potent inhibitor of mitochondrial function ^84, 85^ and was produced in abundance at sites of inflammation ^93^ suggested that sodium channels and raised internal sodium may also be responsible for degeneration of axons in inflammatory lesions ^55, 94^. This reasoning suggested that axons may be rendered vulnerable to degeneration by impulse conduction because this would promote sodium influx, and the combination of electrical activity and NO exposure was found to be a potent cause of degeneration ^95^. It followed that axons may be protected from degeneration by partial blockade of their sodium channels using pharmacological agents ^96, 97^, and the potency of this therapeutic approach was demonstrated in a number of investigations in EAE by using phenytoin ^98^, flecainide ^99^, lamotrigine ^100^, carbamazepine ^101^, and safinamide ^102^ and blockers of the NCX ^97^. Confidence that sodium channel blockade might provide an effective neuroprotective strategy in MS was enhanced by the discovery that the agents could also reduce the severity of inflammation and dampen microglial activation ^102– 106^, and two clinical trials have been performed. The first explored the value of lamotrigine in reducing the rate of brain atrophy in secondary progressive MS ^107^. The trial failed its primary outcome measure, probably because the trial design did not allow for pseudoatrophy resulting from a reduction in inflammatory swelling (which was unexpected at the time), but there was a significant reduction in the deterioration of the secondary outcome measure of the 25-foot timed walk ^107^. Importantly, in the treated group, there was a significant reduction in circulating neurofilament, a marker of neuronal/axonal degeneration ^108^. The second trial examined whether phenytoin was effective in neuroprotection in acute optic neuritis ^109^, and the treated group showed a significant (30%) reduction in the loss of the retinal nerve fibre layer compared with placebo (the trial won the MS Research Prize for 2016).

Of the nine VGSC isoforms identified in mammals, Na _v_1.2 and Na _v_1.6 are predominantly expressed in the axolemma. In myelinated axons, the Na _v_1.6 subtype of VGSCs, which produces both transient and persistent currents ^110– 112^, is strategically located in high density at the nodes of Ranvier ^113^ to allow fast propagation of action potentials. In contrast, Na _v_1.2 VGSCs are preferentially located along the axolemma of unmyelinated axons ^114^. During MS and EAE, the expression of these and other VGSC isoforms is dysregulated ^49, 115– 117^, contributing to ongoing damage and the expression of symptoms ^118, 119^. Thus, it seems reasonable that further understanding the contribution of various VGSC isoforms to pathogenesis and progression in MS could facilitate more targeted therapy, thereby increasing efficacy while reducing any potential side effects. Recently, a non-CNS penetrant sodium channel blocking agent was successful in limiting damage in progressive EAE and a model of optic neuritis ^120^, and the drug gained selective access to inflamed regions of the CNS because of the associated breakdown of the blood-brain barrier. This therapeutic strategy could offer an opportunity to reduce side effects while retaining the beneficial effects of VGSC therapy.

An important adaptation of axons to demyelination in MS and EAE is the expression of both Na _v_1.2 and Na _v_1.6 isoforms along the denuded axolemma ^49, 115^. Whilst these changes allow the restoration of conduction through the lesion ^50– 52, 121^, they may also add a vulnerability to degeneration through the mechanisms described above. The Na _v_1.6 isoform, which is abundantly expressed, has been advanced as being the main mediator of axonal injury because it not only induces a larger persistent sodium current than Na _v_1.2 ^122^ but also frequently co-localises with markers of axonal damage ^49^. More recently, Schattling *et al*. ^123^ showed that a mutation of Na _v_1.2 that results in increased persistent sodium current can also increase degeneration in EAE. Furthermore, the authors showed that genetic manipulation of this isoform had no effect on the immune response in this model, implying that blocking Na _v_1.2 activity may allow neuroprotection without the added immunomodulatory response seen with conventional pan-sodium channel blockers.

Na _v_1.5 was recently reported to be upregulated in astrocytes in both monophasic and chronic-relapsing EAE, significantly correlating with disease severity ^116^. As with microglia, reactive astrocytes can be protective or detrimental; however, their pro-inflammatory effects have been suggested to contribute significantly to the inflammatory response in EAE ^124^, presumably through reactive astrogliosis and glial scar formation. It is noteworthy that Na _v_1.5 has been shown to play an important role in astrogliosis via reverse operation of the NCX and a subsequent robust calcium response *in vitro* ^125^; thus, targeting Na _v_1.5 may represent a therapeutic target for modulating reactive astrogliosis in MS and EAE.

## Conclusions

In recent years, there has been a burgeoning of therapies for MS, most of which interfere with aspects of the acquired immune system. In this review, we have focussed rather on energy balance within the inflamed CNS, taking EAE as an animal model. We have identified tissue hypoxia and free radicals as important factors in the observed mitochondrial dysfunction, and discussed how this can result in neurological dysfunction, sodium dysregulation, calcium entry, and degeneration. Sodium channel inhibitors have unexpectedly emerged as neuroprotective agents, and these deserve more attention as they provide a safe, new therapeutic strategy that may help to redress an energy insufficiency by reducing neuronal energy demand. The other side of the ‘energy coin’, namely increasing energy supply, may be provided by increasing tissue oxygenation, and the first promising observations that this will provide an effective therapy are now emerging.

## Abbreviations

CNS, central nervous system; CypD, cyclophilin D; EAE, experimental autoimmune encephalomyelitis; GSH, glutathione; mPTP, mitochondrial permeability transition pore; MRI, magnetic resonance imaging; MS, multiple sclerosis; NADPH, nicotinamide adenine dinucleotide phosphate; Na-K, sodium-potassium; NCX, sodium-calcium exchanger; NO, nitric oxide; NOX, nicotinamide adenine dinucleotide phosphate oxidase; Nrf2, nuclear factor (erythroid-derived 2)-like 2; ROS/RNS, reactive oxygen/nitrogen species; SWI, susceptibility-weighted imaging; VGSC, voltage-gated sodium channel.
